# A repeated cross‐sectional analysis of the Icelandic baby food market surveyed in 2016, 2019 and 2021

**DOI:** 10.1111/mcn.13476

**Published:** 2023-02-03

**Authors:** Birna Thorisdottir, Tinna Odinsdottir, Inga Thorsdottir

**Affiliations:** ^1^ Faculty of Food Science and Nutrition, School of Health Sciences University of Iceland Reykjavik Iceland; ^2^ Health Science Institute, School of Health Sciences University of Iceland Reykjavik Iceland

**Keywords:** complementary feeding, food environment, food policy, infant and child nutrition, marketing, public health, sugars

## Abstract

World Health Organisation (WHO) has stated that countries need to know their local commercial baby food (CBF) market. Data from other countries suggest rapid changes in CBF options, highlighting the need for repeated analysis. In that context, this repeated cross‐sectional study analysed the options and nutrient quality of different CBF types available in Iceland in years 2016, 2019 and 2021. Data was gathered on formulas, porridge flours, foods in jars and pouches, finger‐foods, other CBF. They were classified into 26 subgroups based on ingredients and taste (sweet/savoury). Minimum consumer age as suggested by the manufacturers and nutritional content were registered. In each data‐collection, 250–275 products were available. Over a third of products (37%–44%) were in pouches. Availability of products intended for 4–11‐month‐old infants decreased, driven largely by a 65% decrease in availability of food in jars (sweet/savoury) between 2016 and 2021. Availability of products intended from 12+ months or without age‐labels increased, driven largely by quadrupling of finger‐foods (predominantly sweet) between 2016 and 2021. The overall percentage of products classified as sweet increased from 65% (2016) to 73% (2019) and 77% (2021). Some finger‐foods had high sugar content (up to 72 g/100 g), partly from fruit concentrate or sugar/syrup. Like other countries, the Icelandic CBF market has moved towards less availability of food intended in the first year and more availability of sweet finger‐foods for an expanded consumer age. As sugar is added to some CBF, stronger regulations on promotion of foods for young consumers and updated recommendations for parents/caregivers may be needed.

## Key messages

- Commercial baby foods (CBF), often monotonous in taste and texture and of differing nutritional quality, are widely available in food retail establishments.
- World Health Organisation has stated that countries need to know their local CBF market.
- Our results show added sugar in some products marketed towards young children and highlights the public health importance of identifying products that can and cannot be promoted for infants and young children.
- Parents/caregivers may need guidance on how to navigate the increasingly complex CBF market to feed children a balanced diet supporting growth, development, evolving nutritional requirements and feeding skills, and establishment of food habits.

## Graphical abstract

This repeated cross‐sectional study analysed the options and nutrient quality of different commercial baby food available in food retail in Iceland in years 2016, 2019 and 2021. Changes in the composition of available foods occurred, mostly driven by decreased availability of food in jars intended for ages below 12 months and increased availability of sweet finger‐food intended for 12+ months or without an age label. Stronger regulations on promotion of foods for young consumers and updated recommendations for parents/caregivers reflecting rapid changes may be needed.

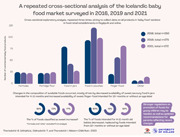

## 1 INTRODUCTION

Complementary feeding, that is the transition from exclusively breast milk and/or infant formula feeding to the consumption of the varied family diet, typically lasts from around 4–6 months until around 2 years of age (White et al., 2017), despite the global recommendation of exclusive breastfeeding until 6 months (WHO & UNICEF, 2003). This is a critical period of growth, development, evolving nutritional requirements, evolving feeding skills, and establishment of food preferences (EFSA Panel on Nutrition, Novel Foods and Food Allergens et al., 2019; Mura Paroche et al., 2017; White et al., 2017). To establish healthy dietary habits, infants need ample opportunities to learn to consume and familiarise them with healthy foods by repeated exposure to different tastes, textures and appearances and by imitating parents' or other role models' eating behaviours (Mura Paroche et al., 2017).

Globally, however, inadequate quality of complementary foods, insufficiently diverse diets and poor feeding practices threaten children's nutrition and health (White et al., 2017). A recent systematic review on infant feeding interventions to prevent childhood obesity highlighted that in contrast to breastfeeding and introduction of solid foods, modifiable parental and environmental factors relating to infant feeding have received little attention in research (Matvienko‐Sikar et al., 2019). Among those factors is the food environment, including availability and nutrition quality of commercial baby food (CBF), that can influence parental food choice for their babies and thereby their diets (Caspi et al., 2012; WHO Regional Office for Europe, 2019a, 2019b). The dynamics of the CBF market are important for policymakers, health authorities and practitioners, as well as industry and consumers. The World Health Organisation (WHO) has stated that it is important for countries to know which CBF are in the market, including the types of food and the nutrient quality, and furthermore, that parents and caregivers need guidance on how to navigate the CBF market to feed children a balanced diet (WHO Regional Office for Europe, 2019a, 2019b). The current study on a local CBF market adds to the knowledge provided by important surveys from around the world (da Rocha et al., 2021; Garcia et al., 2020; Maalouf et al., 2017; WHO Regional Office for Europe, 2019a, 2019b, 2021), and is to our knowledge the first to explore changes between three surveys conducted at different times in the same setting, using the same method. The Icelandic infant nutrition recommendations were revised in 2017 and only briefly mentioned some types of CBFs. However, the options in the market were expected to have changed since then, in line with for example the UK baby food market (Garcia et al., 2020), highlighting the importance of repeated monitoring and publication of the data.

We performed a cross‐sectional exploratory study, repeated three‐times, with the aim to describe the commercial baby food market in Iceland in 2016, 2019 and 2021. Changes in availability of foods according to type, taste, and minimum consumer age were analysed as well as the nutritional content of foods available in 2016. Additionally, the nutritional content of foods classified as finger‐foods available in 2019 and 2021 is described.

## 2 METHODS

### 2.1 Data collection

In October–November 2016, November 2019, and November 2021, the same researcher (TO) gathered information on all commercial baby foods available in‐store in supermarkets and grocery stores (*n* = 1–3 individual stores from *n* = 6 chains at all time‐points and an additional one in 2016 which thereafter left the market), health stores and drug stores in Reykjavik Capital Area (*n* = 4 individual stores at all time‐points), and Icelandic online grocery stores (*n* = 2 in addition to online stores of supermarkets also covered in‐store). Data collection was restricted to foods and fluids in the “baby food” sections and liquids specifically marked for infants and toddlers up to 24 months in the milk cooler. Vitamin D drops and other supplements were excluded from the data collection. The researcher attempted to include all CBF available at these three time‐points.

Information on each product was obtained by photographing information on food packaging in‐store and from information provided by online grocery stores. For each product, the following data was registered: product name, manufacturer's name, recommended minimum age of consumers according to manufacturer. Further, for all products in 2016 and selected products in 2019 and 2021 (see below), information was registered on ingredients and nutritional content. All data was entered into a database in Microsoft Excel.

### 2.2 Classification into food categories

The CBF were classified into six major food categories according to characteristics: formulas (either ready‐to‐drink or powder to be mixed with water), porridge flours (cereal flour to be mixed with water, formula or milk), foods in jars (ready‐to‐eat food in glass jars, plastic packs or cans typically to be consumed with spoon), foods in pouches (ready‐to‐eat food in squeeze food pouches typically to be consumed straight from the pouch through a plastic nozzle), finger‐foods (ready‐to‐eat dry pieces of food typically to be unpacked and given to child for self‐feeding), and “other” CBF products (not fitting into the other groups).

### 2.3 Classification into subgroups based on ingredients and taste

Formulas were further classified as infant formulas (for infants up to 6 months of age) and follow‐on formulas (for infants from 6 months of age) and on whether they were liquid or a powder. The other food categories were further classified into subgroups based on ingredients and taste (sweet or savoury). Porridge flour was classified as flavoured (with fruit, sweet) and non‐flavoured (savoury) and on whether it was iron fortified. Foods in jars and pouches were classified according to their main ingredient as suggested by the name and confirmed by the product label as fruit purées (sweet), vegetable purées (savoury), fruit and vegetable purées (sweet), savoury meals either vegetarian (including for example rice, pasta, potatoes, pulses, cheese, or a combination of those) or including meat or fish, yogurt with fruit (sweet), and porridge either with fruit (sweet) or vegetables (savoury). Finger‐foods were classified according to ingredients into 4 groups. Fruit gummy (sweet) contained mainly fruit and/or fruit concentrate (fruit purée and/or fruit juice concentrate), with most often zero but a maximum of 5% flour, flour not being among the first 3 ingredients. Typical names were in line of fruit bars/bites/melts/snack/sticks/. Fruit or vegetable crackers had >10% flour that was among the first 3 ingredients. Fruit crackers (sweet) contained fruit or fruit concentrate, very often at least partly from apples, with the sweet ingredient >10% and/or listed in among the first 3 ingredients. Veggie crackers (savoury) had <10% fruit/fruit concentrate that was not among the first 3 ingredients. Typical names of crackers were in line of either fruit or vegetable (or lentil, corn or rice) bakes, bars, biscotti, biscuits, cakes, puffs, sticks, or wafers. Breakfast cereals (sweet) came with the suggestion on packaging to pour milk over before consumption and contained sugar or fruits. The remaining “other” products in the baby food sections were juices, grains (quinoa) and oils.

### 2.4 Classification into age‐group

The recommended minimum age of consumers as suggested by the manufacturers was categorised into the following age‐groups that were chosen in the context of milestones in infant feeding: from birth, 4–5 months (early introduction of complementary foods), 6 months (recommended start of complementary feeding), 7–11 months, 12+ months. When consumer age was not specified on the packaging or manufacturer's website, but the product was nevertheless situated in the baby food sections and clearly marketed towards young children below 24 months, the product was categorised as having no age label.

### 2.5 Information on ingredients and nutritional content

In 2016, information on ingredients were collected from all product labels and the nutritional content of each product was collected from the nutrition facts label, complemented, when possible, by more detailed nutrition information on manufacture's websites. This was done for the primary purpose of constructing a commercial baby food composition database to be used in dietary studies of infants and young children in Iceland. Since the availability of finger‐foods expanded between 2016 and 2019, a decision was made in 2019 to collect additional information on ingredients and nutritional content for new finger‐foods in the market. This was also done in 2021. Therefore, the nutritional information that follows is from 2016 for all products except finger‐foods, for which it includes all products available in 2016, 2019 and 2021.

For products that needed reconstitution with liquid (e.g., formula powder and porridge flour), nutrition information was registered on the assumption that preparation was done in accordance with manufacturer's instructions. For each product, the following was registered: energy (kilojoules per 100 g), protein (g per 100 g), carbohydrates (g per 100 g), sugar (g per 100 g), fat (g per 100 g), saturated fatty acids (g per 100 g), and sodium (g per 100 g).

### 2.6 Data analysis

Statistical analysis was performed with R: A Language and Environment for Statistical Computing (R Core Team, R Foundation for Statistical Computing, Vienna, Austria, 2021, https://wwwR-project.org). Descriptive statistics were used, and data presented as n, percentages (%), medians, 25th and 75th percentiles.

## 3 RESULTS

In the three data‐collections, information was registered for a total of 473 CBF products (34 formulas, 41 porridge flours, 94 foods in jars, 179 food pouches, 106 finger‐foods, and 19 “other”) from 33 manufacturers. Around half of both products and producers were in the market in only one data‐collection (55% and 48% in one, 23% and 12% in two, 22% and 40% in three for products and producers, respectively). Two manufacturers were Icelandic, one with four jars of savoury foods in 2016, and the other MS Iceland Dairies with a liquid follow‐on formula made with Icelandic milk, available continuously since 2003. In 2021, half of all CBF in the market came from four manufacturers: Semper and Coop Änglamark (both Nordic), Ella's kitchen (UK), and HiPP (from mainland Europe).

### 3.1 Changes in availability

The total number of CBF increased by 9% from 2016 (*n* = 250) to 2021 (*n* = 272) and changes in the types of available products occurred (Table 1). Availability of food in jars decreased, from representing nearly one‐third of the total CBF products in 2016 to only 10% in 2021, and availability of finger‐foods increased, from representing less than 10% of the total CBF products in 2016 to 30% in 2021. The number of foods in jars decreased by 65% while the number of finger‐foods increased almost fourfold. The number of formulas and porridge flours remained quite stable between 2016 and 2021, with most formulas being powdered and about half of porridge being iron‐fortified. The number of foods in pouches, which was the largest CBF category all 3 years, spiked in 2019, mainly due to a large selection of sweet fruit purée, but was quite similar in 2016 and 2021.

**Table 1:** Availability of commercial baby foods in Iceland

Food category and subgroups	Taste classification	Year 2016, *n*	Year 2019, *n*	Year 2021, *n*
Formulas		25 (10%)	24 (8%)	23 (8%)
Infant formula	None	15	15	14
Follow‐on formula	None	10	9	9
Porridge flours		23 (9%)	23 (8%)	25 (9%)
Non‐flavoured, iron fortified	Savoury	6	9	7
Non‐flavoured, unfortified	Savoury	7	5	7
Flavoured, iron fortified	Sweet	5	7	7
Flavoured, unfortified	Sweet	5	2	4
Food in jars		80 (32%)	43 (16%)	28 (10%)
Fruit purée	Sweet	28	17	13
Vegetable purée	Savoury	15	7	7
Meat or fish meal	Savoury	25	13	6
Vegetarian meal	Savoury	7	3	2
Yogurt with fruits	Sweet	5	3	0
Food in pouches		94 (38%)	121 (44%)	99 (37%)
Fruit purée	Sweet	50	63	52
Fruit and vegetable purée	Sweet	12	11	9
Vegetable purée	Savoury	4	8	6
Meat or fish meal	Savoury	9	7	5
Vegetarian meal	Savoury	2	3	1
Yogurt with fruits	Sweet	10	14	9
Porridge with fruits	Sweet	7	15	14
Porridge with vegetables	Savoury	0	0	3
Finger‐foods		21 (8%)	57 (21%)	82 (30%)
Fruit gummy	Sweet	10	20	48
Fruit cracker	Sweet	10	23	21
Veggie cracker	Savoury	1	11	11
Breakfast cereal	Sweet	0	3	2
Other CBF		7 (3%)	7 (3%)	15 (6%)
Juice	None	6	6	10
Quinoa	None	0	0	3
Oil	None	1	1	2
Total number of products		250 (100%)	275 (100%)	272 (100%)

*Note*: In parenthesis are the percentages of total products per data‐collection categorised as formulas, porridge flours, food in jars or pouches, finger‐foods and other CBF.

Abbreviation: CBF, commercial baby foods.

Every subgroup of food in jars decreased between data‐collections. The availability of fruit or vegetable purée in jars decreased by 53%, from *n* = 43 in 2016, to *n* = 24 in 2019 and *n* = 20 in 2021. The availability of meals in jars (meat/fish or vegetarian) decreased by 75%, from *n* = 32 in 2016, to *n* = 16 in 2019, and *n* = 8 in 2021. Yogurt in jars disappeared from the market. On the other hand, all subgroups of finger‐foods increased from 2016 to 2019. From 2019 to 2021, however, the availability of fruit gummy continued to increase while other subgroups of finger‐foods remained almost unchanged.

### 3.2 Changes in taste

**Figure 1:**
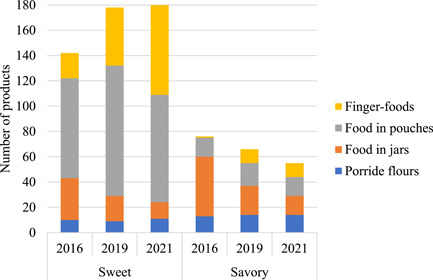
Taste classification of porridge flours, foods in jars, food in pouches and finger‐foods per data‐collection

While over half of porridge flours and food in jars were classified as savoury (57%, 71% and 66% of porridge flours and 59%, 53% and 54% of food in jars, in 2016, 2019 and 2021, respectively), food in pouches and finger‐foods were predominantly sweet (84%, 85% and 85% of food in pouches and 95%, 81% and 87% of finger‐foods, in 2016, 2019 and 2021, respectively). Due to the decreased availability in food in jars and increased availability of finger‐foods, the total % of products classified as sweet increased from 65% in 2016 to 73% in 2019 and 77% in 2021 (Figure 1). In 2021, consumers not restricted by minimum consumer age could choose from 179 sweet and 55 savoury products.

### 3.3 Changes in minimum consumer age

Infant formulas were the only CBF intended for infants from birth up to 4 months (Table 2). All follow‐on formulas were intended from 6 months, except for one product in each data‐collection. Porridge flours were available for different ages from 4 months up to 12+ months, however, with limited choice of different products within age‐groups. For example, consumers wishing to buy iron‐fortified non‐flavoured porridge flour in year 2021 could choose from only *n* = 3 different products labelled from 4 to 5 months, *n* = 1 labelled from 6 months, *n* = 2 labelled from 7 to 11 months, and *n* = 1 labelled from 12+ months. Food in jars and pouches were available for different ages from 4 months up to 12+ months, food in pouches additionally with no age label. Finger‐foods were available for different ages from 6 months, but the majority were labelled for 12+ months or with no age label (76%, 61% and 77% in 2016, 2019 and 2021, respectively).

**Table 2:** Availability of commercial baby foods in Iceland by minimum consumer age according to manufacturer

Minimum consumer age	Year 2016 (*n*)	Year 2019 (*n*)	Year 2021 (*n*)
From birth	15 (6%)	15 (5%)	14 (5%)
Formulas	15	15	14
4–5 months	75 (30%)	75 (27%)	58 (21%)
Porridge flours	10	9	7
Food in jars	35	21	20
Food in pouches	25	42	28
Other	5	3	3
6 months	86 (34%)	92 (33%)	80 (29%)
Formulas	9	8	8
Porridge flours	8	6	11
Food in jars	19	8	3
Food in pouches	50	65	53
Finger‐foods	0	5	5
7–11 months	39 (16%)	37 (13%)	30 (11%)
Formulas	1	1	1
Porridge flours	3	5	5
Food in jars	21	9	4
Food in pouches	9	5	6
Finger‐foods	5	17	14
12+ months	22 (9%)	43 (16%)	41 (15%)
Porridge flours	2	3	2
Food in jars	5	5	1
Food in pouches	0	2	5
Finger‐foods	15	33	30
Other	0	0	3
No age label	13 (5%)	13 (5%)	49 (18%)
Food in pouches	10	7	7
Finger‐foods	1	2	33
Other	2	4	9
Total number of products	250 (100%)	275 (100%)	272 (100%)

*Note*: In parenthesis are the percentages of total products per data‐collection that fall into the respective age category.

Abbreviation: CBF, commercial baby foods.

**Figure 2:**
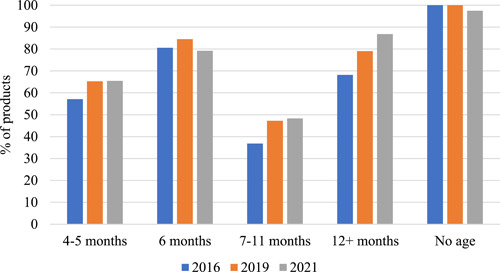
Percentage of the sum of porridge flours, foods in jars, foods in pouches and finger‐foods classified as sweet per data‐collection

The age‐groups 4–5 months and 6 months were the largest in all data‐collections. While the % of products available for younger than 4 months remained the same, the % of foods intended for 4–11‐months‐old infants decreased between data‐collections (from 80% in 2016 to 74% in 2019 and 62% in 2021), replaced by a larger % of foods intended from 12+ months or without an age label (from 14% in 2016 to 21% in 2019 and 33% in 2021). These changes were driven by the decrease in food in jars and increase in finger‐foods. Most products in all age‐groups, except for 7–11 months, were classified as sweet (Figure 2). All products with no age label were sweet, with one exception in 2021.

### 3.4 Ingredients and nutritional content of commercial baby foods

Nutritional content differed within food groups (with noticeable narrow range for formulas) and between food groups, with finger‐foods having by far the highest energy, carbohydrate, and sugar content (Table 3). Porridge prepared from flour according to instructions provided approximately twice as much energy as food in jars or pouches, including meat or fish meals in jars or pouches. Vegetable purée in jars and pouches were noticeable low in energy, while foods in jars or pouches including fruits had on average higher sugars level than other foods in jars or pouches.

**Table 3:** Nutritional content per 100 g of commercial baby foods on the Icelandic market

		Energy (kJ^a^)	Protein (g)	Fat (g)	SFA^b^ (g)	Carbohydrates (g)	Sugars (g)	Sodium (g)
Food groups	*n*	Median (25th–75th percentiles)	Median (25th–75th percentiles)	Median (25th–75th percentiles)	Median (25th–75th percentiles)	Median (25th–75th percentiles)	Median (25th–75th percentiles)	Median (25th–75th percentiles)
Formulas^c^	25	281 (280–288)	1.4 (1.3–1.6)	3.5 (3.3–3.5)	1.2 (1.1–1.4)	7.5 (7.3–8.2)	6.9 (5.6–7.3)	0.02 (0.02–0.03)
Infant formula	15	280 (279–281)	1.4 (1.3–1.6)	3.5 (3.5–3.6)	1.3 (1.2–1.4)	7.3 (7.3–7.5)	7.0 (5.9–7.4)	0.02 (0.02–0.02)
Follow‐on formula	10	290 (283–292)	1.5 (1.4–1.5)	3.2 (2.9–3.3)	1.1 (1.1–1.2)	8.2 (8.2–9.4)	6.7 (5.1–7.2)	0.03 (0.02–0.03)
Porridge from porridge flour^c^	23	536 (496–760)	4.2 (3.7–4.9)	4.0 (2.8–6.0)	1.1 (0.8–2.1)	19.5 (18.0–26.1)	8.1 (6.6–8.7)	0.04 (0.01–0.05)
Non‐flavoured, iron fortified	6	570 (521–600)	4.4 (3.7–5.3)	5.3 (4.3–5.8)	1.2 (1.1–1.2)	17.5 (15.5–18.0)	7.3 (6.2–8.0)	0.05 (0.05–0.06)
Non‐flavoured, unfortified	7	787 (636–791)	4.2 (3.8–4.8)	6.1 (4.3–6.3)	2.2 (1.5–2.2)	26.1 (25.8–28.2)	8.6 (8.3–8.7)	0.00 (0.00–0.00)
Flavoured, iron fortified	5	531 (530–536)	4.5 (4.2–4.8)	3.0 (3.0–3.4)	0.8 (0.8–0.9)	19.5 (19.0–20.0)	7.5 (6.6–8.1)	0.04 (0.04–0.06)
Flavoured, unfortified	5	489 (416–498)	3.7 (3.6–3.7)	2.6 (2.4–3.5)	1.0 (0.8–2.1)	19.0 (13.0–19.0)	8.8 (5.1–9.9)	0.03 (0.03–0.05)
Food in jars^c^	80	278 (219–311)	1.2 (0.5–2.9)	0.7 (0.2–2.5)	0.2 (0.1–0.6)	10.0 (7.8–12.9)	3.1 (2.0–9.4)	0.02 (0.01–0.03)
Fruit purée	28	255 (230–297)	0.5 (0.4–0.6)	0.2 (0.1–0.2)	0.1 (0.0–0.1)	13.3 (11.9–15.3)	10.4 (8.9–11.6)	0.01 (0.00–0.01)
Vegetable purée	15	154 (129–186)	0.8 (0.7–1.2)	0.3 (0.2–1.1)	0.1 (0.0–0.2)	5.8 (4.9–7.4)	2.5 (1.7–3.4)	0.03 (0.01–0.03)
Meat or fish meal	25	305 (292–323)	3.0 (2.8–3.1)	2.6 (2.4–3.0)	0.6 (0.4–0.7)	8.5 (7.5–10.0)	1.9 (1.5–2.3)	0.03 (0.02–0.06)
Vegetarian meal	7	291 (270–313)	3.0 (2.7–3.1)	2.1 (2.0–2.4)	0.7 (0.5–1.0)	8.7 (8.4–9.3)	2.2 (2.1–2.6)	0.03 (0.03–0.04)
Yogurt with fruits	5	330 (292–346)	1.6 (1.4–1.7)	1.1 (0.7–1.4)	0.7 (0.6–0.8)	14.0 (12.0–16.3)	7.9 (7.0–10.7)	0.02 (0.02–0.04)
Food in pouches^c^	94	259 (223–305)	0.7 (0.5–1.3)	0.5 (0.2–1.3)	0.1 (0.0–0.2)	12.3 (10.1–14.3)	10.0 (8.0–12.0)	0.01 (0.00–0.02)
Fruit purée	50	258 (231–279)	0.5 (0.4–0.7)	0.2 (0.1–0.5)	0.1 (0.0–0.1)	13.1 (12.0–15.0)	11.7 (10.2–13.1)	0.01 (0.00–0.02)
Fruit and vegetable purée	12	214 (199–221)	0.6 (0.5–0.8)	0.5 (0.2–0.5)	0.1 (0.1–0.1)	10.1 (9.7–10.8)	9.1 (8.0–9.4)	0.01 (0.01–0.02)
Vegetable purée	4	197 (169–210)	1.4 (1.2–1.4)	1.1 (0.3–1.8)	0.1 (0.1–0.1)	5.7 (4.5–7.3)	2.8 (2.3–3.3)	0.02 (0.01–0.02)
Meat or fish meal	9	298 (251–304)	3.0 (2.8–3.1)	2.8 (1.7–3.0)	0.5 (0.4–0.6)	7.8 (6.2–8.1)	2.0 (1.7–2.3)	0.02 (0.02–0.03)
Vegetarian meal	2	246 (220–273)	2.1 (2.1–2.2)	2.3 (2.0–2.5)	0.8 (0.8–0.8)	6.8 (5.9–7.7)	2.7 (2.5–2.8)	0.04 (0.04–0.05)
Porridge with fruits	7	340 (293–367)	1.0 (1.0–1.2)	1.9 (0.6–2.0)	0.1 (0.1–0.1)	15.0 (13.8–15.5)	8.0 (7.6–9.3)	0.00 (0.00–0.03)
Yogurt with fruits	10	357 (331‐389)	2.1 (1.6–2.6)	1.9 (1.1–3.1)	1.0 (0.6–2.0)	13.2 (12.3–15.5)	11.1 (9.5–11.9)	0.02 (0.01–0.03)
Finger‐foods^d^	106	1523 (1351–1711)	3.1 (1.9–6.8)	2.0 (0.8–11.2)	0.4 (0.1–1.5)	67.2 (62.4–74.0)	36.5 (7.6–59.0)	0.02 (0.00–0.04)
Fruit gummy	55	1352 (1161–1388)	1.9 (1.8–2.5)	0.9 (0.2–1.3)	0.2 (0.0–0.3)	69.0 (63.0–74.0)	59.0 (37.8–67.3)	0.02 (0.00–0.04)
Fruit cracker	31	1675 (1602–1782)	6.4 (5.7–6.9)	11.0 (8.9–13.9)	1.5 (1.1–3.7)	68.3 (63.5–73.5)	16.5 (6.8–30.4)	0.03 (0.01–0.06)
Veggie cracker	17	1797 (1658–1894)	8.5 (7.0–8.7)	14.0 (11.0–17.2)	1.7 (1.1–1.9)	64.8 (61.0–70.0)	2.7 (0.7–3.6)	0.01 (0.00–0.13)
Breakfast cereal	3	1600 (1567–1616)	10.0 (9.8–10.5)	2.9 (2.7–4.2)	0.8 (0.7–0.9)	74.0 (69.4–76.0)	12.0 (12.0–15.4)	0.05 (0.04–0.05)
Other^c^	7	177 (174–245)	0.1 (0.1–0.3)	0.1 (0.1–0.3)	0.1 (0.1–0.3)	10.0 (8.8–10.8)	9.0 (7.1–10.0)	0.03 (0.02–0.04)
Juice	6	177 (172–192)	0.2 (0.1–0.3)	0.1 (0.0–0.1)	0.1 (0.0–0.1)	10.0 (9.6–11.1)	9.0 (7.6–10.5)	0.03 (0.02–0.04)
Oil	1	3700	0.0	100.0	9.1	0.0	0.0	0.00

^a^ Kilojoules, kJ.

^b^ Saturated fatty acids, SFA.

^c^ Nutritional content of products available in 2016.

^d^ Nutritional content of products available in 2016, 2019 and 2021.

All types of finger‐foods were high in carbohydrates (>60 g per 100 g). Fruit and veggie crackers (two of the finger‐food subgroups) often contained >50% flour, with veggie crackers being a little higher in energy, protein, and fat and with lower content of sugars. The finger‐food subgroup fruit gummy had noticeably high sugar content (median 59 g per 100 g), with the highest being 72 g sugar per 100 g. Among common ingredients in fruit gummy was fruit concentrate and in a few products sugar or syrup.

### 3.5 Observation

Some of the finger‐foods without a specified age label were clearly marketed towards children with figures on the packaging such as the puppies from Paw Patrol or Peppa Pig in bright colours, statements of “no added sugar” or “no additives” and encouragements such as being suitable to introduce kids to fruit and vegetables or to increase kids' consumption of fruit and vegetables. However, some of them also had sentences such as “healthier snack for school and after school” and “ideal for kids and adults.”

## 4 DISCUSSION

Today's parents in Western societies have the pleasure, or face the overwhelming task, depending on the point of view, of navigating a market of many hundred different baby food products. While our Icelandic population of 376,000 people can choose from close to 300 CBF, similar to Denmark, Spain and Israel in 2016–2018 (WHO Regional Office for Europe, 2019a, 2019b), parents in some other European countries, the United States and Brazil may choose from around 800 up to a few thousand CBF (da Rocha et al., 2021; Garcia et al., 2020; Maalouf et al., 2017; WHO Regional Office for Europe, 2019a, 2019b, 2021). Not only the total number of products, but also the different properties and nutritional quality of products, rapid changes and inappropriate promotion of some commercial foods make the situation complex (Grammatikaki & Caldeira, 2019; WHO Regional Office for Europe, 2019b). Being advised to exclusively breastfeed until 6 months while having access to a wide variety of CBF labelled as suitable for infants from 4 to 5 months may be confusing for parents. This exposure may even potentially signal parents that early introduction of complementary foods may be beneficial. Great care needs to be taken to not undermine breastfeeding (Grummer‐Strawn et al., 2017; Harris & Pomeranz, 2020; WHO, 1981).

While breastfeeding and consumption of diverse home‐made foods is emphasised for infants and toddlers, CBF are recommended in some circumstances (Grammatikaki & Caldeira, 2019). If an infant is not exclusively breastfed in the first 6 months, commercial infant formula is the recommended substitute for breast milk (Bagci Bosi et al., 2016; WHO & UNICEF, 2003). Icelandic guidelines, the most recent ones from 2017, further recommend commercial follow‐on formula if another milk than breast milk is consumed between 6 and 24 months and porridge made from iron‐fortified, non‐flavoured commercial porridge flour from start of complementary feeding until at least 9–12 months of age (Directorate of Health, 2017). These recommendations are based on robust evidence on the importance during complementary feeding of adequate iron intake (Bates et al., 2020; Domellöf et al., 2014; Thorisdottir et al., 2011, 2013), and not too high protein intake (Ferré et al., 2021; Koletzko et al., 2009; Patro‐Gołąb et al., 2016; Thorisdottir et al., 2013). Formulas and iron‐fortified, non‐flavoured porridge flours together only make up 12% of the CBF in Iceland. Since the minimum age of intended consumers affects the nutritional content and texture of porridge flour, our findings of limited choice per age‐group suggest that following the recommendation regarding iron‐fortified, non‐flavoured porridge flours result in limited diversity in brands and textures.

The Icelandic guidelines also mention variable nutritional quality of CBF in jars and pouches and encourage parents to scrutinise ingredient lists and nutrition labels. The convenience of these products under special circumstances is acknowledged while it is stressed that they are not recommended for every‐day use (CBF in jars) or except very occasionally (CBF in pouches) (Directorate of Health, 2017). While frequent consumption of any CBF can result in the child being exposed to monotonous taste and texture of food, the sucking of purée from pouches additionally results in missed opportunities for learning about the smell and look of food, training of spoon‐feeding, and active participation of a caregiver in feeding the child, all of which are important factors in establishing healthy food habits that may affect nutrition and health (EFSA Panel on Nutrition, Novel Foods and Food Allergens et al., 2019; Fewtrell et al., 2017; Matvienko‐Sikar et al., 2019; Mura Paroche et al., 2017; UNICEF, 2021). Therefore, we consider the greatly decreased availability of CBF in jars since 2016, which may result in parents rather choosing purées or meals in pouches, an undesirable development. This seems to be a global, or at least Western trend seen for example in the United Kingdom and United States (Beauregard et al., 2019; Business Research Company, 2022; Garcia et al., 2020).

Another likely global trend is the increasing availability of commercial finger‐foods marketed for babies. In the United Kingdom, availability of finger‐foods increased by 440% from 2013 to 2019 (Garcia et al., 2020), very similar to our findings of 390% increase between 2016 and 2021. It is very important to note the different nutritional properties of these products compared to other CBF. They are often very high in energy, carbohydrates, and sugars. Some of the finger‐foods (the subgroup fruit gummy) had similar ingredients and nutritional properties as gummy bears or similar candy. Also, highly important to note is that most finger‐foods were labelled for 12+ months (37%) or were without an age label (40%). This needs to be further investigated in our local market but it can be expected that some of the finger‐foods without an age label should ideally not be situated in the “baby food” sections of stores or otherwise marketed for infants and toddlers (WHO Regional Office for Europe, 2019b). WHO even recommends that some products should be specifically labelled as not suitable for infants and young children up to 3 years (WHO Regional Office for Europe, 2019b). Locating these products in the “baby food” section next to formulas, porridge flours and savoury foods in jars, for example, complicates matters greatly for consumers that need to be very alert when choosing products for their young ones as this section is not at all a “safe to buy for infants and young children” area. Commercial finger‐foods are not mentioned in the current Icelandic infant nutrition recommendations, likely due to the small market share during the last update of the recommendations (Directorate of Health, 2017) but our findings give good reasons for revising them as soon as possible to guide parents and other caregivers on how to navigate the increasingly complex CBF market in Iceland.

### 4.1 Strength and limitations

The main strength of the study is the careful data collection where a trained researcher, always the same one and always the same time of the year, gathered information using the same pre‐specified methodology. Since the Icelandic CBF market is relatively small compared to many other countries, we are in key position to keep monitoring the local market. Further, given the similar changes seen in our study as in the United Kingdom for example (Garcia et al., 2020), our monitoring may have the potential to invoke policy changes in addition to guiding consumers on how to choose CBF as part of a healthy, balanced diet of infants and young children. We attempted to obtain a comprehensive overview of CBF on the Icelandic market but acknowledge that there is a possibility of missing or misclassifying individual products. We consider the nutritional values of good quality, since all nutritional values were given for ready‐to‐eat products with the exception of formulas and porridge flour, for which good preparation instructions were available.

The main limitation is that this is a survey of the local market in a single country with a small population. Despite this, we consider it an important contribution of global interest for policymakers and researchers since evidence on the status and development of CBF markets in different countries can be of value in identifying priorities in public health interventions and policy making. As earlier discussed, the overall size of our CBF market is similar to some larger countries and some of the trends identified seem to be global trends. Readily available open‐source evidence seems to be lacking from other Nordic countries (Denmark, Finland, Norway, Sweden). Another limitation is that a more comprehensive methodology for assessing the promotion and marketing of products was not used (WHO Regional Office for Europe, 2019a, 2019b), which can be explained by the primary aim of the 2016 data collection which was to construct a new Icelandic Commercial Baby Food Composition Database and the continuation of the methodology constructed at that time. Combined with the Icelandic Food Composition Database (ISGEM) it is the base on which dietary intake of infants and young children in Iceland is calculated. The Icelandic Commercial Baby Food Composition Database is currently being updated for finger‐foods as the nutritional values of the 21 products from 2016 were not representative of the total sum of 106 products that have been in the market from 2016 to 2021. Thereafter, intake of CBF will be calculated from 3‐day food records kept at 6, 9, 12 and 24 months for around 200 Icelandic participants. If repeating the data‐collection at a later point, we would follow the more comprehensive methodology currently being developed and tested in many countries (WHO Regional Office for Europe, 2019a, 2019b, 2021).

### 4.2 Conclusions

This study revealed important changes in the Icelandic commercial baby food market between data‐collections in 2016, 2019, and 2021, moving from an emphasis on food mainly intended during complementary feeding, largely requiring active parental involvement in feeding, towards sweet finger‐foods for self‐feeding of an expanded consumer group of children older than 1–2 years, even up to school age or longer. We argue that the quality of available CBF may be decreasing and raise questions about the ethical aspect of locating CBF without an age label, including sweet finger‐foods containing fruit purée, fruit juice concentrate or even sugar or syrup, in the baby food sections of food retail establishments. Showing that sugar is added to some products marketed towards young children highlights the public health importance of identifying products that can and cannot be promoted for infants and young children, as advised by the WHO (WHO Regional Office for Europe, 2019b). We further recommend an update of guidance on complementary feeding practices in Iceland and likely some other countries to reflect the modern context of increasingly complex CBF markets.

### AUTHOR CONTRIBUTIONS

**Birna Thorisdottir and Tinna Odinsdottir**: formulated the research questions and designed the study. **Tinna Odinsdottir**: performed the field work and database work. **Birna Thorisdottir** and **Tinna Odinsdottir**: drafted the paper. **Birna Thorisdottir, Tinna Odinsdottir**, and **Inga Thorsdottir**, and **Inga Thorsdottir**: contributed analysing the data and writing the article. All authors have read and accepted the final version of the article.

## CONFLICTS OF INTEREST

The authors declare no conflicts of interest.

## Data Availability

The data that support the findings of this study are available from the corresponding author upon reasonable request.
